# Probiotic Almond-Fermented Beverages Processed by Ultrasound: Vegan and Non-Vegan Consumer Perceptions through Packaging

**DOI:** 10.3390/foods13131975

**Published:** 2024-06-22

**Authors:** Gabrielly Ribeiro Carneiro, Caique dos Santos Rocha, Mariana Vitória Pardim Fernandes, Carlos Eduardo Barão, Tatiana Colombo Pimentel

**Affiliations:** 1 Department of Food Engineering, State University of Maringá (UEM), Maringá 87020-900, Paraná, Brazil; gabriellyrcarneiro@gmail.com (G.R.C.); caique.rocha300@gmail.com (C.d.S.R.); 2 Federal Institute of Paraná, Campus Paranavaí, Paranavaí 87703-536, Paraná, Brazil; marianamavi05@gmail.com (M.V.P.F.); carlos.barao@ifpr.edu.br (C.E.B.)

**Keywords:** non-thermal technology, emotional profile, emerging technologies, consumer perception, probiotic non-dairy products, *Dipteryx alata* Vogel

## Abstract

Consumer perception of foods processed by emerging technologies has been scarcely studied. This study aimed to evaluate the perception of vegan and non-vegan consumers regarding probiotic almond-fermented beverages processed by ultrasound using the packaging of the products (pasteurized/conventional, processed by ultrasound, and processed by ultrasound with a claim on the label). A “Check All That Apply” test with emojis and the Food Technology Neophobia scale were used. The “processed by ultrasound” information did not impact the purchase intention and the perception of healthiness, safety, nutrition, environmental impact, flavor, texture, and price of the products. The claim inclusion increased the perceived acceptability and purchase intention and improved the emotional profile. The vegan consumers showed a more positive perception of ultrasound processing, resulting in increased perceived acceptability, higher citation frequency of positive emoji, and lower sums for the neophobia scale. Vegan and non-vegan consumers agreed that the most important attributes for consumer acceptance are almond aroma, flavor, and consistency. In conclusion, the “processed by ultrasound” information did not negatively impact the acceptability and emotional profile of probiotic almond-fermented beverages, and using a claim on the label may improve consumer perception of the products.

## 1. Introduction

In the last decade, the popularity of non-dairy fermented beverages has increased, mainly due to the higher number of lactose intolerants, vegans, vegetarians, and flexi-vegans [1]. Baru almonds (*Dipteryx alata* Vogel) have high nutritional value and considerable concentrations of phenolic compounds [2,3]. In this way, the Baru almond milk may be a raw material for plant-based beverages, and its fermentation with probiotics could be an alternative to obtain a functional non-dairy product [4].

Dairy and non-dairy products are generally thermally treated using pasteurization to ensure a safe product. However, pasteurization can negatively impact products’ sensorial and nutritional quality [5]. Ultrasound is an emerging technology used as an alternative to pasteurization in food products [6]. In our previous study, we observed that the application of ultrasound to Baru almond milk and its utilization in the processing of probiotic fermented beverages reduced fermentation time and resulted in beverages with higher probiotic survival, bioaccessibility of fatty acids and phenolic compounds, and in vitro antidiabetic properties. Furthermore, it improved consistency, stability, volatile composition, sensory properties, and consumer acceptance compared to pasteurized products [7].

Sensory characteristics have been considered essential when choosing food products, and they directly affect the consumer’s perception of the products. However, non-sensory attributes have gained importance in recent years, mainly because they can reflect a consumer’s prior perception, i.e., before a possible acquisition if the product were on the market. In the case of food products processed using emerging technologies, they can be considered unfamiliar and unacceptable at first contact. Consumer rejection can occur for different reasons, such as lack of knowledge about the technology, health, safety, ethical and environmental concerns, and an increase in the product’s price [8,9]. In this sense, identifying barriers and drivers of acceptance of foods processed by emerging technologies requires adequate measurement using validated scales, such as the Food Technology Neophobia scale [10].

Packaging is responsible for the first communication with consumers, and it may impact how consumers perceive the products before purchase and generate emotional responses [11,12]. Therefore, a prior assessment of products in the form in which they would be sold (packaging) can generate important information about products subjected to emerging technologies [13]. In addition, the packaging and information on the label (claims) can impact consumers’ purchasing decisions [11]. However, the consumer perception of foods processed by emerging technologies has been scarcely studied [9,13,14,15].

The emotional profile of a food product can help discriminate between products with similar acceptance, provide information for product development and marketing, and help predict consumer behavior toward a product [16]. Emojis are graphic symbols that have become popular worldwide with the advancement of digital communication technologies [17]. Emojis have the advantage of offering a non-verbal approach to convey meaning that would otherwise not be expressed in words or would be expressed differently [18,19]. Using emojis in evaluating consumers’ emotional responses to food products is a promising approach [19], as people often use emojis in their communications [20]. The emotional profile is constantly evaluated using the Check All that Apply (CATA) test with an emoji list [21].

Vegan and non-vegan consumers may have different perceptions about probiotic non-dairy fermented beverages processed by emerging technologies, which would directly impact marketing strategies [22]. Therefore, this study aimed to evaluate the perception of vegan and non-vegan consumers regarding probiotic almond-fermented beverages processed by ultrasound using the CATA test with an emoji list and the packaging of the products with or without a claim on the label. Furthermore, the beverages’ perceived acceptability (expected liking), healthiness, nutrition, safety, environmental impact, flavor, texture, and price were assessed. In addition, the important sensory attributes for consumer acceptance were determined. Finally, the Food Technology Neophobia scale evaluated the openness to new foods.

## 2. Materials and Methods

### 2.1. Packaging Preparation

**Figure 1:**
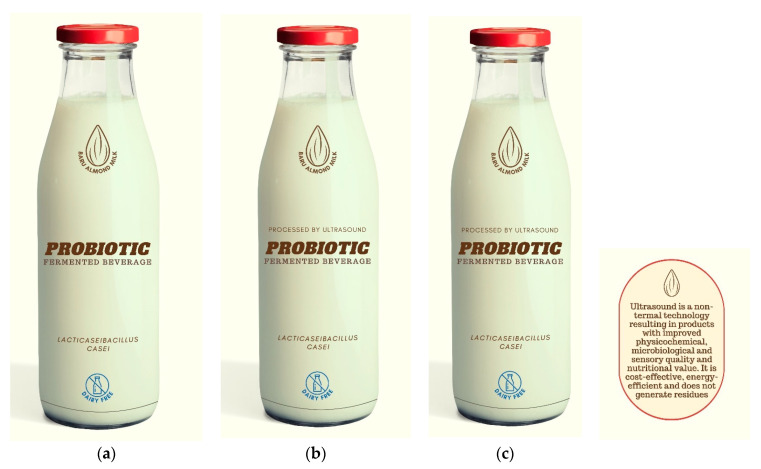
Packaging design. (**a**) conventional processing, (**b**) processed by ultrasound, (**c**) processed by ultrasound, and with a claim.

Digital packaging for probiotic almond-fermented beverages was prepared based on commercially available products and using an online graphic designer (CANVA, Australia, Sydney). The packaging showed information about using Baru almond milk as raw material, the probiotic strain (*Lacticaseibacillus casei*), and the dairy-free symbol (Figure 1). Three types of packaging were prepared. One packaging was based on the conventional beverage (subjected to pasteurization, PAST, Figure 1A), and the second packaging contained information about ultrasound processing (processed by ultrasound, US, Figure 1B). The third packaging contained information about ultrasound processing (processed by ultrasound) and a claim: “Ultrasound is a non-thermal technology resulting in products with improved physicochemical, microbiological, and sensory quality and nutritional value. It is cost-effective, energy-efficient, and does not generate residues” (US-CLAIM, Figure 1C). The pasteurized product did not have the “pasteurized” information because it is not needed for commercialization, as it is the conventional processing. The ultrasound claim was constructed based on previous studies that evaluated the application of ultrasound in food processing [7,23,24,25].

### 2.2. Recruitment of Consumers and Questionnaire Application

This study was approved by the Ethics Committee of Human Beings of the State University of Maringá (Approval number: 38348920.0.0000.0104). An online questionnaire was constructed on Google Forms and tested by the project members and selected people to make necessary corrections. After finalization, the questionnaire was distributed online through social media like Facebook, Instagram, and Twitter. The link and description of the research were available for access. To obtain participants from the vegan group, the questionnaire was also shared in the Facebook groups: Veganoser (3.8 thousand participants), Veganos Rio (6.5 thousand participants), Vegetarians and Vegans (8.1 thousand participants), Vegetarian and Vegan Diet (67.8 thousand participants), Vegetarians and Beginner Vegans Brazil (49 thousand participants), among others. The inclusion criteria for the study were to be older than 18 years old, Brazilian, and available to participate in the test. No restrictions regarding gender, residence place, or education level were applied. We did not screen the consumers to select only those who consume plant-based fermented beverages. We aimed to obtain a general perspective of consumer perceptions and increase the availability and consumption of these products in the Brazilian market.

The questionnaire initially asked for sociodemographic information (gender, age, education level), the diet type (vegan or not), and consumption of dairy (yogurt and fermented milk) and non-dairy (coconut, almond, oat) fermented beverages (Table 1). Six hundred respondents (n = 600), 300 non-vegans and 300 vegans answered the questionnaire. Previous studies have used a similar number of participants when evaluating the consumer perception of food products [4,11,14,26,27]. The participants included predominantly women (70%) aged between 18 and 35 years old (56.7–73.7%) and with a bachelor’s degree or above (80%). Non-vegan consumers had a high frequency of consumption of yogurts and fermented milk (74.4% consumed at least once a month). Furthermore, both groups reported relatively high consumption of non-dairy fermented beverages (41.1–47.7% consumed at least sometimes a month) (Table 1).

**Figure 2:**
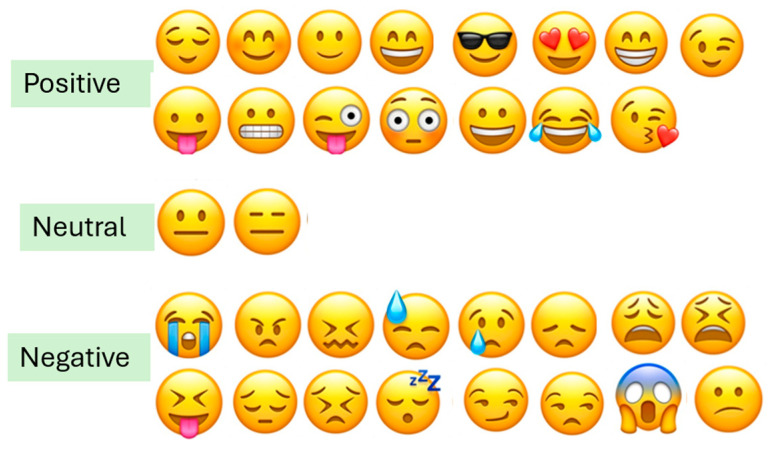
List of emojis [31].

Subsequently, the first packaging was presented, and the participant’s perceptions were assessed using a Check All that Apply (CATA) test containing an emoji list (Figure 2). The emoji used were taken from “EmojiOne” [28], which is an online platform that provides free emoji under a Creative Commons license that complies with Apple’s Unicode standard as described by Gallo et al. [29,30]. The emoji list consisted of 33 emojis, which have been reported as the most used in food studies [31], and they were presented in 3 columns of 11 emoji in the questionnaire.

The participants were instructed to select all emoji describing their emotional experience with the product using the CATA test [16]. The statement “Select all emojis you believe are related to what you have felt once you see this product” was presented. Participants were also asked to answer about perceived acceptability (expected liking) using a 9-point hedonic scale (1 = disliked very much, 9 = liked very much). Furthermore, healthiness, safety, nutrition, environmental impact, flavor, texture, and price perceptions were evaluated using a 7-point Likert scale (1 = totally disagree; 4 = neither agree nor disagree; 7 = totally agree). For that, the participants received the following statements: “Based on the image, I believe the product is healthy”, “Based on the image, I believe the product is safe”, “Based on the image, I believe the product is nutritive”, “Based on the image, I believe the product benefits the environment”, “Based on the image, I believe the product is tasty”, “Based on the image, I believe the product has a suitable texture”, and “Based on the image, I believe the product has an accessible price”. Furthermore, the purchase intention was evaluated using a 5-point scale (1 = would certainly not buy; 3 = maybe buy, maybe not buy, 5 = would certainly buy) [11,21]. Then, the second and third packaging were evaluated using the same procedures. For randomization of the 3 types of packaging, 6 different questionnaires were prepared, each answered by 50 consumers of each group (vegan and non-vegan) in a completely randomized design. The emojis were randomized in the list for each participant, selecting this feature in Google Forms.

After the packaging evaluation, the respondents were asked to answer the Food Technology Neophobia scale questions using a 7-point Likert scale (1 = totally disagree; 4 = neither agree nor disagree; 7 = totally agree) (Table 2). This scale evaluates the new food openness and consists of thirteen (n = 13) sentences [10,32,33].

Furthermore, the importance of attributes of probiotic fermented beverages for consumer acceptance was evaluated using a 7-point Likert scale (1 = not important; 7 = very important) [9]. The attributes evaluated were fermented beverage appearance, acid aroma, almond aroma, almond flavor, fermented beverage flavor, and consistency. These attributes were selected from our previous study with probiotic Baru almond-fermented beverages [4]. For that, the consumers were asked, “For you, how important is that the probiotic almond-fermented beverage you consume has a fermented beverage appearance?”, “For you, how important is that the probiotic almond-fermented beverage you consume has an acid aroma?”, “For you, how important is that the probiotic almond-fermented beverage you consume has an almond aroma?”, “For you, how important is that the probiotic almond-fermented beverage you consume has almond flavor?”, “For you, how important is that the probiotic almond-fermented beverage you consume has fermented beverage flavor?”, and “For you, how important is that the probiotic almond-fermented beverage you consume is consistent?”.

### 2.3. Statistical Analysis

For the CATA data (emoji list), a Cochran test and McNemar (Bonferroni) test were performed to assess differences in emoji frequency citations between packaging for each group (vegan and non-vegan consumers). Furthermore, a Correspondence Analysis (CA) map was constructed using a matrix of 900 rows (300 consumers and 3 packagings) and 33 columns (33 emojis) for each group (vegan or non-vegan consumers) [20]. A multiple-factor analysis (MFA) was performed to visualize the relationship between the emotional profile provided by the consumer groups (vegan or non-vegan). The MFA was obtained using a matrix of 3 rows (3 packaging) and 34 columns (17 significant emojis for the two groups) [27]. The RV coefficient and significance were obtained [34].

Data on perceived acceptability, healthiness, safety, environmental impact, flavor, texture, price, and purchase intention were subjected to Analysis of Variance (ANOVA) and Tukey’s mean comparison test for comparisons among packaging (*p* < 0.05). For evaluating differences between consumer groups (vegan and non-vegan consumers), a Student’s *t*-test for independent samples was applied (*p* < 0.05) [27].

For the Food Technology Neophobia scale and attributes important for acceptance, the mean and standard deviation were calculated. Furthermore, factor analysis (FA) was performed using Principal Component Analysis (PCA) as the extraction method, with factors with eigenvalues greater than 1 considered relevant. The analysis was carried out using Varimax rotation. Kaiser-Meyer-Olkin (KMO) and Cronbach’s alpha coefficient tests were obtained and used to evaluate the confidence level [9]. All statistical analyses were performed using XLSTAT 2022.2 software (Adinsoft, Paris, France).

## 3. Results and Discussion

### 3.1. Perceived Acceptability (Expected Liking) and Purchase Intention

The results of the perceived acceptability (expected liking) and purchase intention evaluations of the probiotic almond-fermented beverage packaging are shown in Table 3. The perceived acceptability of the products was between 7.39 and 7.89 for non-vegans and 7.93 and 8.55 for vegans on a 9-point hedonic scale. Furthermore, the purchase intention was between 3.84 and 4.00 for non-vegans and 4.07 and 4.33 for vegans on a 5-point scale. Therefore, the consumers liked the products moderately to much and probably would buy them.

A higher perceived acceptability and purchase intention was observed for the vegan consumers (*p* < 0.05). These results may be associated with this group’s higher familiarization and consumption of plant-based beverages (Table 1). Familiarity may increase the personal experience of purchasing and consuming foods and the ability to conceptualize them, resulting in increased acceptance and purchase intention [34]. Furthermore, consumers may have different motivations and attitudes depending on the diet type [35].

Differences were observed between formulations for both groups (*p* < 0.05). For the non-vegan consumers, the information “processed by ultrasound” did not impact the perceived acceptability and purchase intention (*p* > 0.05). At the same time, the claim inclusion increased the perceived acceptability and purchase intention (*p* < 0.05). For vegan consumers, both applying the US and claim inclusion increased the perceived acceptability (*p* < 0.05), while only the claim increased the purchase intention (*p* < 0.05). Our results demonstrate that including the “processed by ultrasound” information did not impact (non-vegans) or improve (vegans) the products’ perceived acceptability compared to the conventional product. Therefore, vegan consumers were more prone to accept the US as a novel technology for processing fermented beverages.

The similar or better-perceived acceptability of the US compared to pasteurized products is an important result, as previous studies have reported negative impacts of emerging technologies on consumer acceptance [13,14]. Generally, consumer acceptance of products subjected to emerging technologies relies on providing relevant information about the technology, resulting in greater expected acceptance [1,14], corroborating our result that the claim inclusion increased the perceived acceptability and purchase intention for both groups. Consumers look for information about the product and want to be convinced that they are making an adequate choice when choosing the product subjected to the emerging technology and not the conventional one [14]. In this way, claim inclusion may be an essential motivator to increase the expected acceptability and the purchasing intention of products subjected to emerging technologies [15].

### 3.2. Emotional Profile by CATA with Emoji List

Table 4 presents the contingency table for the CATA data for non-vegan and vegan consumers. In general, the consumers had a positive perception of the products, as positive emojis were used by both groups (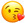, 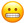, 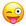, 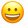, 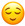, 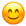, 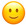, 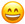, 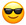, 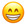, and 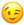), and negative emojis were not used by both groups (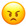, 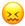, 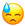, 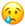, 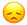, 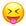, 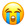, 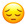, 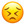, 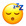, 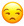, 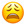, and 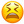). These results corroborate those of perceived acceptability (Table 3).

**Figure 3:**
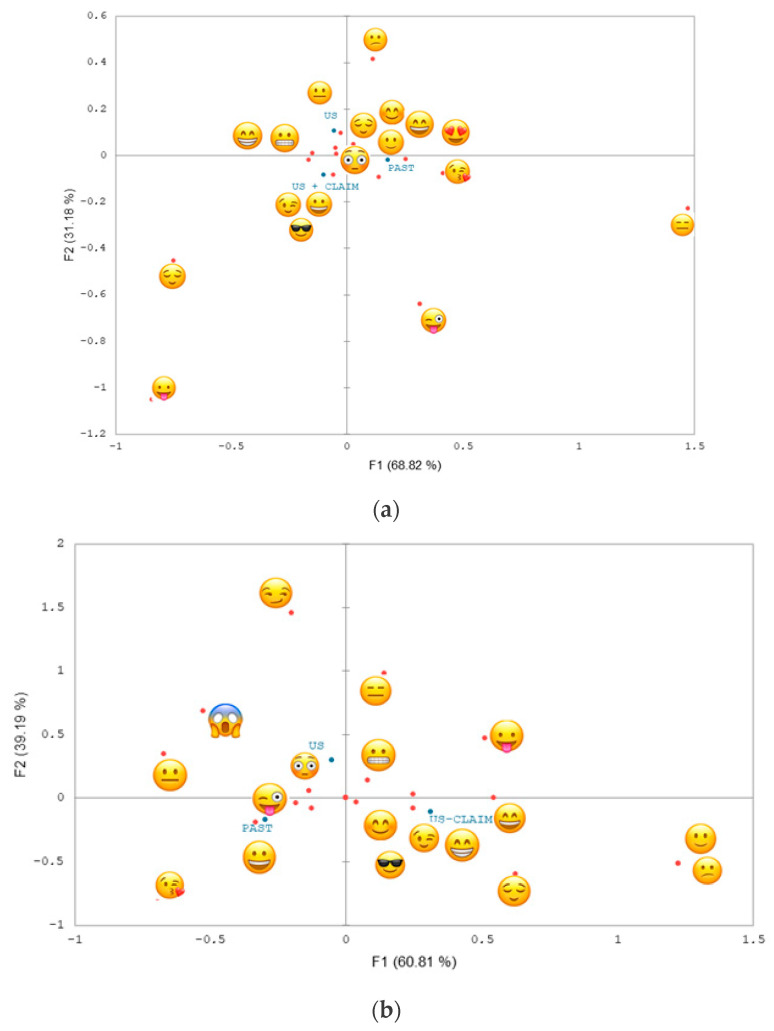
Correspondence analysis for (**a**) non-vegan and (**b**) vegan consumers for CATA data.

For the non-vegan consumers, the first two dimensions of the correspondence analysis (CA) on the frequency table explained 100.00% of the inertia (Figure 3A), and most of the explanation was observed for the first dimension (68.82%). The total inertia explanation is similar to that reported in previous studies with untrained (>90%) [16,20] and trained assessors [36]. The PAST formulation was characterized by the proximity from the 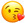, the US formulation from 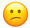, 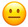, 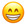, and the US-CLAIM from 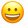, 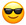, 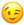, and 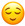.

The non-vegan consumers used 18 emojis to describe the emotional profile of the probiotic almond-fermented beverages, and the most used emojis were 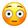 (50%), 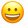 (39%), 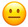 (28%), 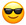 (28%), 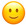 (26%), and 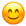 (22%)). They checked 2–21 emojis, with an average of 8.45 emojis. From the emoji list, 6 showed significant differences among formulations (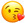, 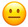, 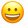, 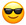, 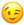, and 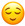, *p* < 0.05, Table 4).

The PAST and US formulations showed a similar citation frequency for 31 out of 33 emoji, demonstrating their similar perception by non-vegan consumers (*p* > 0.05) and corroborating the similar perceived acceptability and purchase intention (Table 3). However, the PAST formulation had a higher citation frequency of 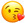, and the US had a higher citation frequency of 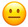 (*p* < 0.05). Therefore, although consumers may not be used to ultrasound technology (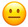), it was perceived as positive and similar to the pasteurized product (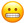, 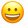, 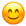, 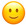, 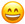, 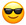, 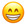, and 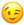). The results interest food industries, as consumers demonstrated no rejection or fear of ultrasound. Our results indicate that evaluating emotional profiles could distinguish formulations with similar perceived acceptability scores. This denotes the importance of using the emotional profile as a complementary approach to acceptance tests to evaluate consumer perception.

Finally, the US-CLAIM formulation showed a higher citation frequency of 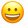, 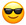, 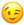 than PAST (*p* < 0.05) and a higher citation frequency of 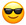 than the US (*p* < 0.05). Finally, the presence of a claim reduced the citation frequency of 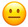 and increased that of 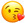 (*p* < 0.05), resulting in similar citation frequencies to PAST (*p* > 0.05). Our results demonstrated that the claim inclusion reduced the doubt about US processing, resulting in a more positive perception of the products. Information about the advantages of emerging technologies may be useful in developing more positive attitudes [13]. Furthermore, informing people about using low temperatures may decrease the perceived risks and increase the perceived benefits [15].

For the vegan consumers, the first two dimensions of the CA on the frequency table explained 100.00% of the inertia (Figure 3B), and most of the explanation was observed for the first dimension (60.81%). The PAST formulation was characterized by the proximity from the 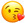 and 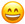, the US formulation from 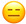, 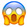, 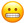, and 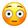, and the US-CLAIM from 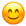, 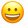, 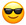, and 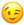.

The vegan consumers also used 18 emojis to describe the emotional profile of the probiotic almond-fermented beverages, and the most used emojis were 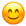 (48%), 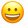 (45%), 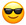 (42%), 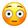 (28%), 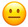 (22%), and 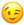 (22%)). They checked 0–20 emoji with an average of 7.45. From the emoji list,14 showed significant differences among formulations (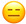, 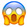, 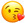, 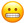, 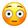, 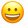, 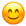, 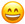, 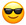, 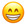, 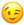, 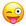, 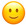 and 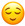, *p* < 0.05, Table 4).

The PAST formulation had a higher citation frequency of 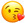, 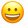, 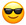, and 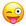. The US had a higher citation frequency of 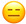, 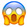, 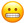, 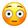, 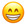, 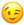 than PAST (*p* < 0.05). These results corroborate those of perceived acceptability (Table 3). In this way, although vegan consumers also demonstrated some lack of knowledge about the technology (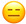), they showed curiosity (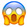, 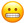) and were more accessible to the US processing, which resulted in higher citation frequencies of some positive emoji for this formulation (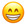, 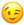). Finally, the US-CLAIM formulation showed a higher frequency of citation of 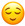, 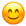, 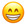, 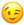, and 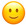 (*p* < 0.05). Furthermore, the presence of a claim reduced the citation frequency of 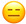 and 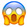 (*p* < 0.05), resulting in similar citation frequencies to PAST (*p* > 0.05). Therefore, similar to the observed for the non-vegan consumers, the claim inclusion could reduce the doubting feeling of the US processing and result in a more positive perception of the products.

We could observe that the claim used in the present study was informative and understandable for both groups of consumers and resulted in positive emotions about the products. However, it is well known that industries consider labeling products a barrier to applying emerging technologies to food products, mainly because it could negatively impact consumers [15]. In our study, including “processing by ultrasound” in the packaging did not negatively impact the emotional profile of the products. Furthermore, the claim inclusion contributed to a more positive emotional profile.

**Figure 4:**
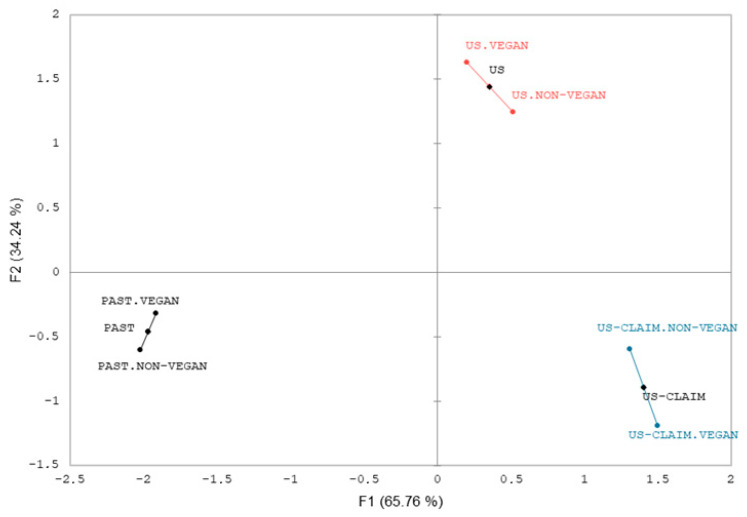
Multiple-Factor Analysis (MFA) for CATA data.

Vegan and non-vegan consumers used the same number of emojis (n = 18), and the most used emojis were similar between groups (Table 4). Figure 4 shows the MFA map for both groups that evaluated the probiotic almond-fermented beverage packaging. The first component (F1) explained 65.76% of the data variability, and the second component (F2) explained 34.24%, totaling 100.00%. The fermented beverage packaging was separated in the maps for both consumer groups, and the projective points for the two groups were close. In the MFA, the projective point proximity indicates the similarity between the groups [34]. The RV coefficient of the map was 0.983 (*p* < 0.0001). The RV coefficient can be used to provide numerical data on the level of similarity between MFA maps [34]. The results indicate similarities between the groups for the different probiotic almond-fermented beverages. This way, both consumers (vegan and non-vegan) have a similar perception of the products, considering their emotional profile. However, it is worth mentioning that this similarity does not necessarily mean that the packaging was differentiated for the same emojis [36], which explains the differences between consumer groups (CA maps and Cochran test and McNemar (Bonferroni) test, Figure 3, Table 4). This result is important because marketing strategies could be used for both vegan and non-vegan consumers. The results suggest that the industries may design the packaging of a product subjected to emerging technologies that could elicit similar emotional experiences and be accepted by both vegans and non-vegans. Furthermore, the advertisements may use the same strategy, covering both publics.

### 3.3. Healthiness, Safety, Nutrition, Environmental Impact, Flavor, Texture, and Price Perceptions

Table 3 presents the results for healthiness, safety, nutrition, environmental impact, flavor, texture, and price perceptions. Both groups moderately agreed that the probiotic almond-fermented beverages were healthy (6.05–6.60), safe (6.02–6.66), and nutritive (6.00–6.51). Furthermore, they slightly agreed that the products benefit the environment (5.51–6.31), are tasty (5.77–6.20), and have suitable texture (5.91–6.20). Finally, they neither agree nor disagree that the products have an accessible price (4.18–4.83). Our results demonstrate that consumers positively perceived the probiotic almond-fermented beverage by considering the health, safety, nutrition, environment, and sensory aspects (taste and texture).

Generally, plant-based beverages are considered healthy and sustainable [37]. However, our results are of paramount importance considering the sensory aspects, as previous studies have reported that non-vegan consumers perceive vegetarian/vegan products as less tasty and with worse texture because they expect these products to be similar to the dairy ones [35,37]. However, the consumers were unsure if the products could be considered at an accessible price. In fact, the retail price of plant-based alternatives is generally higher than their dairy counterparts [38,39]. Furthermore, many Brazilian consumers have reported not buying plant-based beverages due to the high price [37].

A higher healthiness, safety, nutrition, environmental impact, and flavor perception was observed for vegan consumers (*p* < 0.05). Furthermore, they showed a lower price perception (*p* < 0.05), indicating that they consider the price less accessible than the non-vegan consumers. It is well known that vegan consumers are more concerned about sustainability and health [35] and have a more positive perception of non-dairy products. The price impact may be associated with buying these products more constantly than non-vegan consumers and being more aware of the prices. Esperança et al. [37] reported that consumers with high consumption of these products pointed out the high cost of plant-based beverages.

The “processed by ultrasound” information had no impact on healthiness, safety, nutrition, environmental impact, flavor, texture, and price perceptions, regardless of the group (*p* > 0.05). These results corroborate non-vegan consumers’ expected acceptability and emotional profile (Table 3). For vegan consumers, our results suggest that the increased expected acceptability (Table 3) was related to the curiosity and positive emotions provided by US (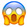, 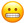, 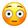, 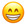, 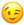) and not to a perceived improvement in healthiness, safety, nutrition, environmental impact, texture, or price promoted by the technology. This way, evaluating the emotional profile was essential to understand the consumer perception of the vegan public.

The claim inclusion positively impacted healthiness, nutrition, environmental impact, and texture for non-vegan consumers and healthiness, safety, nutrition, environmental impact, flavor, and texture for vegan consumers (*p* < 0.05). Food is a significant part of lifestyle, and nutritional properties are a broader concept of well-being and contribute to consumer happiness and satisfaction [15]. The claim may have positively impacted the participants’ perception of the evaluated attributes [15], which corroborates the results of emotional profile (higher frequency citation of positive emojis). These results demonstrate that vegan consumers were more prone to believe in the claims than non-vegan consumers, resulting in positive perceptions. Studies have revealed that not all consumers are convinced after reading the claims, and some may often express skepticism about the benefits of using an emerging technology [13].

### 3.4. Food Neophobia Scale and Attributes Important for Acceptance

Table 2 presents the results of the Food Technology Neophobia scale and attributes important for acceptance. Cronbach’s alfa values were 0.63 and 0.76 for non-vegans and 0.78 and 0.74 for vegans regarding the Food Technology Neophobia scale and attributes important for acceptance, respectively. Furthermore, the KMO was 0.67 and 0.76 for non-vegans and 0.67 and 0.60 for vegans regarding the Food Technology Neophobia scale and attributes important for acceptance, respectively. These results prove that the FA was suitable for evaluating the data.

The sum of scores for the Food Technology Neophobia scale for Brazilian consumers was 51.61 for non-vegans and 47.74 for vegans, similar to the observed for Canadians (58.5) [40] and Australians (55) [32]. Hiver values for the sum of scores indicate less receptivity to new food technologies [9]. In this way, we can note that Brazilian consumers were receptive to new technologies, and vegan consumers were more receptive than non-vegan consumers. These results corroborate those of the products’ perceived acceptability and emotional profile, as US-treated products showed increased perceived acceptability and higher citation frequencies of positive emoji by vegan consumers (Table 3 and Table 4).

The first two FA dimensions explained 46.59% and 52.40% for non-vegans and 56.99% and 67.08% for vegans, considering the Food Technology Neophobia scale and attributes important for acceptance, respectively. Relatively low explanation values have been reported when the assessors were consumers, as there are high variations in the use of the scales by them [9,10,15].

The first FA dimension was correlated with 1–5 and 9–12 items of the neophobia questionnaire for both groups of consumers. Therefore, consumers believe that “new technologies are necessary” (items 1–5 and 12), healthier options (item 7), and without risk (items 9–11) [33]. Furthermore, the second FA was correlated with 6–8 items of the neophobia questionnaire for both groups of consumers. Item 13 was associated with the first FA for non-vegans and the second FA for vegans.

The consumers had a positive perception of emerging technologies. They considered them necessary, as they disagreed that society should not depend too heavily on technology to solve its food problem (statement 11) and that there is no sense in trying out high-tech food products because the ones I eat are already good enough (statement 12). Furthermore, they agree that they could increase the product availability on the market. They agreed that new technologies used in food production and/or processing give people more control over their food choices (statement 7) and disagreed that there are already numerous tasty foods on the market, so we do not need new food technologies to produce more (statement 4).

At the same time, the consumers demonstrated a positive perception of food technologies on the quality, healthiness, environmental impact, and safety of food products. They disagreed that new technologies used in the production and/or processing of food decrease the natural quality of food (statement 5), may have long-term negative environmental effects (statement 9), that it can be risky to switch too quickly to new technologies in the production and/or processing of food (statement 10), and that new foods are not healthier than traditional foods (statement 2). However, they are unaware of the impact on health in the long term, as they neither agree nor disagree that new technologies used in food production and/or processing probably would not bring negative health effects in the long term (statement 4).

We could observe that the main limitation of new food technologies was related to the lack of information and/or distrust in the media. The consumers of both groups agreed that they are not fully familiar with new technologies employed in producing and/or processing food (statement 1), neither agreed nor disagreed that the statements about the benefits of new technologies used in the production and/or processing of foods are often grossly overstated (statement 3) and disagreed that the media usually provides a balanced and unbiased view of the new technologies employed in food production and/or processing (statement 13).

Our results demonstrate that Brazilian consumers are open and receptive to new food technologies. No signs of rejection or fear were observed from the answers, mainly regarding possible sensory changes, environmental impact, or safety risks. However, doubts are present regarding the long-term effects of these technologies on health. In this way, understandable, trustable, and clear information should be provided by industries to overcome the lack of confidence [9]. Finally, it is important to state the drawbacks of the technology so the consumers may not believe that there are overstated benefits.

The first FA dimension was correlated with 3–4 items for the non-vegans and 1, 2, and 5 items for vegans, considering the attributes important for the acceptance questionnaire. At the same time, the second FA dimension was correlated with 1, 2, and 5 items for non-vegan consumers and 3–4 items for vegan consumers. The consumers of both groups agreed with all statements (scores approximately or higher than 5), denoting that fermented beverage appearance and flavor, acid aroma, almond aroma and flavor, and consistency are important attributes of the probiotic almond-fermented beverage acceptance. However, items 3, 4, and 6 showed a higher mean value, demonstrating that almond aroma and almond flavor and consistency may be the most important attributes to be considered for both groups of consumers. These results could be used by industries considering the critical attributes of plant-based fermented beverages. Therefore, the application of emerging technologies should maintain or improve these attributes.

The main limitation of this study is that the consumers of both groups were mainly females, young, and with a high level of education. Gender, age, income, and education level are some of the factors that influence food choices [41]. Generally, the younger generations are less conservative regarding their food choices and tend to be more open-minded to novel food processing [42]. Regarding gender, a previous systematic review reported no differences in food neophobia between men and women [43]. Finally, higher education levels have been associated with lower food neophobia scores [43]. Further studies may evaluate the effect of these aspects on the consumer perception of foods subjected to emerging technologies.

## 4. Conclusions

This study was the first to evaluate the perception of vegan and non-vegan consumers regarding probiotic almond-fermented beverages processed by ultrasound using the packaging of the products (pasteurized/conventional, processed by ultrasound, and processed by ultrasound with a claim on the label). “Processed by ultrasound” information did not impact the purchase intention and the perception of healthiness, safety, nutrition, environmental impact, flavor, texture, and price of the products. The claim inclusion increased the perceived acceptability and purchase intention and improved the emotional profile. The vegan consumers showed a more positive perception of ultrasound processing, resulting in increased perceived acceptability, higher citation frequency of positive emoji, and lower sums for the neophobia scale. Vegan and non-vegan consumers agreed that the most important attributes for consumer acceptance are almond aroma, flavor, and consistency. In conclusion, the “processed by ultrasound” information did not negatively impact the acceptability and emotional profile of probiotic almond-fermented beverages, and using a claim on the label may improve consumer perception of the products.

## Figures and Tables

**Table 1:** Sociodemographic characteristics of the participants (n = 300 for each group: vegans and non-vegans).

Parameter %	Non-Vegans	Vegans
*Gender*		
Female	71.7	77.7
Male	28.3	22.3
*Age (years)*		
18–25	34	10.7
26–35	39.7	46.0
36–45	12.6	23.0
46–55	11.3	6.7
56–65	1.7	
>60	0.7	2.3
*Level of education*		
Below Bachelor’s degree	19.7	17.3
Bachelor’s degree or above	80.3	82.7
*Consumption frequency of yogurts and fermented milk*		
Never	5.3	86.3
Rarely (1–3 times a year)	30.3	13.7
Seldom (sometimes a month)	26.0	0
Often (sometimes a week)	32.0	0
Always (sometime a day)	6.4	0
*Consumption frequency of fermented beverages (coconut, almond, oat)*		
Never	19.6	18.66
Rarely (1–3 times a year)	39.3	33.67
Seldom (sometimes a month)	27.7	17
Often (sometimes times a week)	10.7	21.67
Always (sometime a day)	2.7	9.0

**Table 2:** Food Neophobia Scale and attributes important for acceptance answers.

	Evaluated Parameter	Non-Vegan Consumers			Vegan Consumers		
Question Number		Mean Values	D1	D2	Mean Values	D1	D2
	*Neophobia*						
1	I am not fully familiar with new technologies employed in producing and/or processing food	5.57 ± 1.63	**0.315**	0.024	5.04 ± 1.78	**0.504**	−0.059
2	New foods are not healthier than traditional foods	3.82 ± 1.86	**0.582**	0.062	3.13 ± 2.09	**0.648**	−0.262
3	The statements about the benefits of new technologies used in the production and/or processing of foods are often grossly overstated	3.91 ± 1.88	**0.648**	0.012	4.25 ± 1.99	**0.778**	−0.149
4	There are already numerous tasty foods on the market, so we do not need new food technologies to produce more	2.11 ± 1.78	**0.798**	0.158	1.69 ± 1.29	**0.815**	0.283
5	New technologies used in the production and/or processing of food decrease the natural quality of food	3.28 ± 1.94	**0.745**	−0.047	2.60 ± 1.85	**0.792**	−0.105
6	New technologies used in food production and/or processing will probably not have negative health effects in the long term.	4.47 ± 1.69	0.162	**0.450**	3.85 ± 1.76	0.083	**0.313**
7	New technologies used in food production and/or processing give people more control over their food choices	5.43 ± 1.39	−0.094	**0.992**	5.26 ± 1.70	−0.348	**0.656**
8	New products using new food technologies can help people to have a balanced diet.	5.65 ± 1.32	−0.087	**0.612**	5.62 ± 1.35	−0.435	**0.783**
9	New food production and/or processing technologies may have long-term negative environmental effects.	3.71 ± 1.63	**0.730**	−0.078	3.68 ± 1.50	**0.863**	−0.021
10	It can be risky to switch too quickly to new technologies in the production and/or processing of food	3.60 ± 1.79	**0.702**	−0.118	3.41 ± 1.93	**0.641**	0.065
11	Society shall not depend too heavily on technology to solve its food problems.	3.78 ± 2.08	**0.602**	−0.228	3.86 ± 2.05	**0.735**	−0.288
12	There is no sense in trying out high-tech food products because the ones I eat are already good enough.	2.33 ± 1.77	**0.861**	0.057	2.10 ± 1.61	**0.783**	0.065
13	The media usually provides a balanced and unbiased view of the new technologies employed in food production and/or processing	3.94 ± 1.84	**0.389**	0.123	3.95 ± 1.90	0.075	**0.436**
	Sum of scale	51.61			47.74		
	*Attributes for acceptance*						
1	For you, how important is that the probiotic almond-fermented beverage you consume has a fermented beverage appearance?	5.80 ± 1.32	0.179	**0.690**	5.05 ± 1.74	**0.905**	−0.132
2	For you, how important is it that the probiotic almond-fermented beverage you consume has an acid aroma?	4.87 ± 1.46	0.172	**0.498**	5.15 ± 1.81	**0.668**	0.178
3	For you, how important is that the probiotic almond-fermented beverage you consume has an almond aroma?	5.98 ± 1.41	**0.949**	0.236	5.37 ± 1.41	0.115	**0.993**
4	For you, how important is it that the probiotic almond-fermented beverage you consume has an almond flavor?	6.08 ± 1.39	**0.887**	0.220	5.43 ± 1.46	0.021	**0.840**
5	For you, how important is it that the probiotic almond-fermented beverage you consume has a fermented beverage flavor?	5.43 ± 1.44	0.073	**0.756**	4.91 ± 2.04	**0.802**	−0.035
6	For you, how important is that the probiotic almond-fermented beverage you consume is consistent?	6.04 ± 1.17	0.246	**0.453**	5.83 ± 1.46	**0.544**	0.251

Attributes values were expressed as mean ± standard deviation. Results for 300 consumers of each group (non-vegan and vegan) and based on a 7-point Likert scale: 1 = totally disagree and 7 = totally agree for neophobia scale. Results based on a 7-point Likert scale for sensory attributes (1 = not important, 7 = very important). Significant factor loadings are in bolded in the axis after rotation. D1 and D2 are the factor analysis dimensions after Varimax rotation.

**Table 3:** Acceptability, purchase intention, healthiness, safety, nutrition, environmental impact, flavor, texture, and price perceptions for non-vegan and vegan consumers.

Parameter	Non-Vegan Consumers			Vegan Consumers		
	PAST	US	US-CLAIM	PAST	US	US-CLAIM
Perceived acceptability	7.39 ± 1.40 ^bB^	7.49 ± 1.37 ^bB^	7.89 ± 1.23 ^aB^	7.93 ± 1.00 ^cA^	8.24 ± 0.98 ^bA^	8.55 ± 0.66 ^aA^
Purchase intention	3.84 ± 0.87 ^bB^	3.87 ± 0.92 ^bB^	4.00 ± 0.86 ^aB^	4.07 ± 0.75 ^bA^	4.10 ± 0.77 ^bA^	4.33 ± 0.77 ^aA^
Healthiness	6.10 ± 1.13 ^abB^	6.05 ± 1.05 ^bB^	6.29 ± 0.96 ^aB^	6.19 ± 0.82 ^bA^	6.16 ± 0.94 ^bA^	6.60 ± 0.57 ^aA^
Safety	6.06 ± 1.15 ^aB^	6.02 ± 1.06 ^aB^	6.18 ± 1.02 ^aB^	6.32 ± 0.78 ^bA^	6.21 ± 1.06 ^bA^	6.66 ± 0.64 ^aA^
Nutrition	6.00 ± 1.15 ^bB^	6.00 ± 1.06 ^bB^	6.26 ± 1.02 ^aB^	6.07 ± 1.14 ^bA^	6.14 ± 1.01 ^bA^	6.51 ± 0.69 ^aA^
Environmental impact	5.51 ± 1.36 ^bB^	5.53 ± 1.34 ^bB^	5.85 ± 1.29 ^aB^	5.85 ± 1.06 ^bA^	5.94 ± 1.12 ^bA^	6.31 ± 1.02 ^aA^
Flavor	5.77 ± 1.33 ^aB^	5.79 ± 1.28 ^aB^	5.93 ± 1.18 ^aB^	5.98 ± 1.14 ^bA^	5.97 ± 1.00 ^bA^	6.20± 0.96 ^aA^
Texture	5.97 ± 1.32 ^abA^	6.00 ± 1.19 ^bA^	6.05 ± 1.20 ^aA^	5.99 ± 1.15 ^abA^	5.91 ± 1.06 ^bA^	6.20 ± 0.96 ^aA^
Price	4.59 ± 1.59 ^aA^	4.67 ± 1.60 ^aA^	4.83 ± 1.77 ^aA^	4.18 ± 1.76 ^aB^	4.33 ± 1.79 ^aB^	4.21 ± 1.86 ^aB^

Means ± standard deviation followed by different lowercase letters in the line indicate differences between formulations for the same group (non-vegan or vegan consumers) using the Tukey test (*p* < 0.05). Means ± standard deviation followed by different uppercase letters in the line indicate differences between groups (non-vegan or vegan consumers) for the same parameter using a *t*-test (*p* < 0.05). 9-point hedonic scale for perceived acceptability (1 = disliked extremely, 9 = liked extremely), 5-point scale for purchase intention (1 = certainly would not buy, 5 = certainly would buy), and 7-point Likert scales for healthiness, safety, nutrition, environmental impact, flavor, texture, and price (1 = totally disagree, 7 = totally agree).

**Table 4:** Contingency table for the CATA data on the emoji list for non-vegan and vegan consumers.

		Non-Vegan Consumers				Vegan Consumers			
Emoji Number	Emoji	PAST	US	US-CLAIM	*p*-Value	PAST	US	US-CLAIM	*p*-Value
1	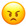	0	0	0	1.000	0	0	0	1.000
2	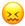	0	0	0	1.000	0	0	0	1.000
3	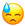	0	0	0	1.000	0	0	0	1.000
4	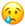	0	0	0	1.000	0	0	0	1.000
5	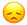	0	0	0	1.000	0	0	0	1.000
6	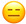	2	0	0	0.135	0 ^b^	19 ^a^	6 ^b^	**<0.0001**
7	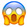	0	0	0	1.000	6 ^b^	12 ^a^	0 ^b^	**0.002**
8	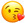	37 ^a^	17 ^b^	19 ^ab^	**0.003**	24 ^a^	0 ^b^	6 ^b^	**<0.0001**
9	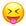	0	0	0	1.000	0	0	0	1.000
10	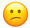	4	6	2	0.223	0	0	7	0.050
11	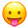	0	0	2	0.135	0	6	6	0.050
12	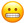	24	17	22	0.444	19 ^b^	20 ^a^	37 ^a^	**0.002**
13	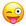	2	0	2	0.135	12 ^a^	6 ^b^	6 ^b^	**0.002**
14	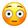	150	139	158	0.189	65 ^b^	96 ^a^	95 ^a^	**<0.0001**
15	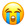	0	0	0	1.000	0	0	0	1.000
16	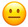	73 ^b^	95 ^a^	82 ^ab^	**0.013**	70	69	57	0.288
17	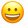	102 ^b^	121 ^ab^	129 ^a^	**0.017**	162 ^a^	125 ^b^	115 ^b^	**<0.0001**
18	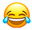	0	0	0	1.000	0	0	0	1.000
19	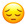	0	0	0	1.000	0	0	0	1.000
20	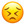	0	0	0	1.000	0	0	0	1.000
21	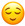	0 ^b^	2 ^b^	6 ^a^	**0.030**	6 ^b^	0 ^b^	1 8^a^	**<0.0001**
22	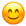	150	164	154	0.331	137 ^b^	129 ^b^	166 ^a^	**<0.0001**
23	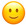	66	82	83	0.148	0 ^b^	0 ^b^	6 ^a^	**0.002**
24	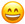	47	44	46	0.937	13 ^a^	0 ^b^	14 ^a^	**0.001**
25	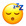	0	0	0	1.000	0	0	0	1.000
26	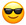	73 ^b^	77 ^b^	102 ^a^	**0.001**	147 ^a^	108 ^b^	120 ^b^	**<0.0001**
27	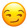	3	2	2	0.867	0	6	0	0.050
28	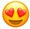	2	0	2	0.368	0	0	0	1.000
29	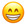	14	21	23	0.164	13 ^c^	32 ^b^	68 ^a^	**<0.0001**
30	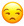	0	0	0	1.000	0	0	0	1.000
31	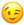	39 ^b^	58 ^ab^	68 ^a^	**0.001**	44 ^c^	63 ^b^	93 ^a^	**<0.0001**
32	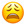	0	0	0	1.000	0	0	0	1.000
33	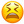	0	0	0	1.000	0	0	0	1.000

Citation frequencies followed by different lowercase letters in the line indicate differences between formulations for the same group (non-vegan or vegan consumers) using the Cochran test and McNemar (Bonferroni) test (*p* < 0.05). *p*-values in bolding are significant.

## Data Availability

The original contributions presented in the study are included in the article, further inquiries can be directed to the corresponding author.
